# Network analysis of affect, emotion regulation, psychological capital, and resilience among Chinese males during the late stage of the COVID-19 pandemic

**DOI:** 10.3389/fpubh.2023.1144420

**Published:** 2023-03-27

**Authors:** Zhihua Guo, Yi Cui, Tianqi Yang, Xufeng Liu, Hongliang Lu, Yinling Zhang, Xia Zhu

**Affiliations:** ^1^Department of Military Medical Psychology, Air Force Medical University, Xi’an, China; ^2^Department of Nursing, Air Force Medical University, Xi’an, China

**Keywords:** positive and negative affect, emotion regulation, psychological capital, psychological resilience, network analysis, COVID-19 pandemic

## Abstract

**Background:**

Previous studies have confirmed that both affect and emotion regulation strategies are closely associated with psychological capital (PsyCap) and resilience. These factors are assumed to buffer the effect of the COVID-19 pandemic on mental health, especially among males. However, these interactions have not been closely examined to date. To fill this gap, this paper explores the dimension-level relationships of these psychological constructs among Chinese males during the late stage of the COVID-19 pandemic and identified critical bridge dimensions using network analysis.

**Methods:**

A total of 1,490 Chinese males aged 21–51 years completed self-report scales assessing emotion regulation strategies, affect, PsyCap, and psychological resilience. Two regularized partial correlation networks, namely the affect and emotion regulation-PsyCap network and the affect and emotion regulation-psychological resilience network, were then constructed to examine links between the dimensions of these constructs. The bridge expected influence (BEI) index was also calculated for each node to identify important bridge nodes.

**Results:**

Positive affect, negative affect, cognitive reappraisal, and expressive suppression showed distinct and complex links to various dimensions of PsyCap or psychological resilience. In both networks, positive affect, cognitive reappraisal, and negative affect were identified as critical bridge nodes, with the first two having positive BEI values and the third having a negative value.

**Conclusion:**

The findings elucidate the specific role of the dimensions of emotion regulation or affect in relation to PsyCap and psychological resilience, which facilitates further understanding of the mechanisms underlying these interrelationships. These findings also provide implications for developing effective intervention strategies to increase PsyCap and psychological resilience.

## 1. Introduction

The coronavirus disease-2019 (COVID-19) pandemic has imposed great challenges on the world. Restrictions on social interactions, increased economic burden, and uncertainty about the future caused by the pandemic have seriously impacted the mental health of the general population (1–4). Notably, gender had an effect on the mental health outcomes resulting from COVID-19—males are susceptible to the negative effects of the pandemic to a certain extent. Having to work from home following a COVID-19 outbreak has been linked to worse mental health in men, but not in women (5). COVID-19 adversely influences male fertility and sexual health, and the effects are both biologically and mentally (6, 7). Males are also at greater risk of severe COVID-19 infection and death compared to females (8, 9). These features may aggravate the effect of COVID-19 on mental health among males and deplete their psychological resources (10). During such a unique period as the global COVID-19 pandemic, people need sufficient psychological resources to maintain mental health (11), which is especially true for males.

Psychological capital (PsyCap) refers to a psychological resource beyond social and human capital (12) that comprises the four dimensions of self-efficacy, optimism, hope, and resilience (13). PsyCap is assumed to be a protective resource to combat psychological distress (14). Psychological resilience, which is defined as coping, adapting, and thriving in the context of adversity (15) or quick recovery of stable functioning after exposure to trauma (16, 17), helps avoid psychological distress in the case of disasters or disease outbreaks (18–21). PsyCap and psychological resilience thus have important implications for mental health outcomes in the context of the COVID-19 pandemic (22, 23). For example, it has been shown that PsyCap buffered psychological distress and protected against depression and anxiety during the COVID-19 pandemic (11, 24). However, mandatory confinement during the pandemic could cause a significant reduction in PsyCap (25). Previous studies have also identified the protective role of psychological resilience against pandemic-related anxiety, depression, psychological stress, and emotional exhaustion (26–29). Therefore, PsyCap and psychological resilience play important roles for maintaining public mental health during the pandemic.

Understanding how relevant factors impact PsyCap and psychological resilience may contribute to strategies to enhance them, thereby attenuating the negative effects of the pandemic on mental health. The relationships between affect, emotion regulation, and PsyCap, as well as those between affect, emotion regulation, and psychological resilience have received extensive attention. Affect, which comprises positive and negative affect (30, 31), is closely related to PsyCap and psychological resilience (32–35). Emotion regulation refers to the capacity to manage and express emotions (36, 37) and reflects anticipation, reactivity, and adaptation occurring after life events (38). According to the personal resource theory, positive affect is promoted by successful emotion regulation and consequently leads to the accumulation of PsyCap (36). Moreover, effective emotion regulation was found to promote PsyCap and reduce psychological impairment caused by the pandemic (39). Studies have also reported that individuals who can effectively regulate their emotions and experience positive affect more frequently have greater psychological resilience to deal with challenges (34, 40, 41). Furthermore, maladaptive emotion regulation was found to buffer the positive effects of psychological resilience on insomnia (42).

Most previous studies tended to regard PsyCap or psychological resilience as a whole and assessed them *via* summed scores, which masks the heterogeneity and extent of essentiality of the different components of PsyCap or psychological resilience. This approach has hindered the understanding of the detrimental or protective effects of positive and negative affect and various emotion regulation strategies on the distinct dimensions of PsyCap or psychological resilience (43–45). In addition, as the influence of positive and negative affect and different emotion regulation strategies on PsyCap and psychological resilience have not been quantified to date, specific intervention targets to enhance PsyCap or resilience are unknown. Similar concerns have been reported in research on depression: the use of sum-scores to assess depression based on the assumption that depression is a single condition and all its symptoms are equivalent has hampered the identification of biomarkers and more efficacious antidepressants (43, 44, 46). Hence, analysis at a fine-grained level, rather than reliance on sum-scores, is an important way forward.

Network analysis is an emerging data-driven approach that is widely used in psychology and psychopathology to estimate and visualize relevant psychological constructs (47, 48) that can satisfy this research requirement. According to network analysis theory, psychological constructs can be considered as a network emerging from interactions between different components (dimensions or items), in which nodes represent interacting components of constructs and edges represent correlation pathways between these components (49–51). Network analysis can graphically delineate these interactions to provide insights into the psychopathological mechanisms and develop targeted intervention strategies (52). Network analysis can also provide meaningful indices to evaluate the roles the nodes play in the network (53). For example, the bridge expected influence (BEI) index is used to assess the influence of a given node on nodes of other psychological constructs. BEI can help to determine the bridge nodes that play important roles in maintaining the co-occurrence of constructs (54), which may be useful to inform interventions (52, 54–56). Network analysis has been applied in prior studies on psychological resilience. A cross-country network analysis study identified caregiver support during stressful times as the most central factor for adolescent resilience (57). The relationships between coping and resilience, as well as the effects of expressive arts therapy on resilience, have also been studied using network analysis (58, 59). However, at present, network analysis has not been used to examine the fine-grained relationships between affect, emotion regulation, and PsyCap, or between affect, emotion regulation, and psychological resilience.

To address this research gap, the present study used network analysis to examine the dimension-level network structures of affect, emotion regulation, and PsyCap, as well as that of affect, emotion regulation, and psychological resilience. Node BEI indices were calculated to quantitatively evaluate the role of nodes in bridging affect and emotion regulation with PsyCap or psychological resilience. The aims of this study are to (1) explore the fine-grained correlation patterns of affect, emotion regulation, and PsyCap, as well as affect, emotion regulation, and psychological resilience; and (2) identify critical bridge nodes that transmit the positive and negative impacts of affect and emotion regulation on PsyCap and resilience. Studying these relationships during the late stage of the COVID-19 pandemic in particular is of significance for efforts to further understand the mechanisms underlying the links between these relationships and to determine targeted intervention strategies that enhance PsyCap and psychological resilience. This study is necessary because recently published studies support that the mental health issues caused by the COVID-19 pandemic are long-lasting (60–62); the present study provides references for the promotion of mental health to meet both current and future challenges caused by the COVID-19 pandemic.

## 2. Methods

### 2.1. Study design and participants

A multi-center, cross-sectional survey was conducted in five communities distributed in southeastern, western, southern, northern, and central China (i.e., Guangzhou, Kashgar, Kunming, Shenyang, and Wuhan) from March 2021 to August in 2021. The time span chosen for this study is relatively long because different cities had different pandemic prevention and control stages. This study was approved by the Ethics Committee of the First Affiliated Hospital of the Air Force Medical University and carried out in accordance with the Declaration of Helsinki. Before the start of the survey, two researchers were trained using a standardized procedure. For data collection, the researchers used standardized instructions to introduce background, objective, procedures, the voluntary nature of participation, declarations of anonymity and confidentiality, as well as the survey questionnaires for participants. All participants provided informed consent. Subsequently, the participants completed the questionnaires independently, which were retrieved on the spot after completion.

A total of 1,600 participants were recruited by convenience sampling and completed paper and pencil tests (i.e., questionnaires). The questionnaires gathered demographic information (e.g., age, gender, parent structure, and educational attainment) and four valid scales to measure emotion regulation, affect, PsyCap, and psychological resilience. The inclusion criteria were as follows: (1) age ≥18 years; (2) gender: male; (3) normal communication skills; and (4) provision of informed consent. Participants who had recently experienced major life events (e.g., bereavement because of the death of a close relative or friend, major injury/illness, and separation or breakup of a personal intimate relationship) were excluded. None of the respondents experienced the above events. Of the 1,600 participants, 93 did not complete all items and 17 selected the same response for all items. Thus, the final sample was 1,490 participants (mean age = 26, SD = 4.40, range = 21–51 years).

### 2.2. Measures

#### 2.2.1. Emotion regulation questionnaire

The emotion regulation questionnaire (ERQ) was used to assess the frequency with which individuals habitually use cognitive reappraisal or expressive suppression to cope with emotional responses (63). The questionnaire comprises 10 items, including cognitive reappraisal (6 items) and expressive suppression (4 items) factors. ERQ is rated on a 7-point Likert scale ranging from 1 = *strongly disagree* to 7 = *strongly agree*, with higher scores indicating a higher usage frequency of emotion regulation strategies. The Cronbach’s α of ERQ in this study was 0.872.

#### 2.2.2. Positive and negative affect scale

The positive and negative affect scale (PANAS) was used to measure each individual’s emotional experience during the past 1–2 weeks (31). The Chinese version of PANAS was employed (64) which includes a total of 18 items, 9 items each for positive and negative affect subscales. Each item is rated on a 5-point Likert scale ranging from 1 = *very slightly or not at all* to 5 = *extremely*, with higher scores indicating stronger feelings and emotions. The Cronbach’s α values of the positive affect subscale and negative affect subscale in this study were 0.924 and 0.843, respectively.

#### 2.2.3. Positive psychological capital questionnaire

The modified 26-item positive psychological capital questionnaire (PPQ), which includes the four dimensions self-efficacy, resilience, optimism, and hope, was used to evaluate PsyCap (65, 66). Each item is rated on a 7-point Likert scale ranging from 1 = *not at all true* to 7 = *entirely true*, with a higher score indicating higher PsyCap. The Cronbach’s α of PPQ in this study was 0.943, suggesting extremely good internal consistency.

#### 2.2.4. Connor-Davidson resilience scale

The 25-item Connor-Davidson resilience scale (CD-RISC) was used to measure psychological resilience (67). In a prior study, the use of CD-RISC disclosed a three-factor structure of resilience among Chinese adults comprising tenacity, strength, and optimism (68). Each item is rated on a 5-point Likert scale ranging from 0 = *not true at all* to 4 = *true all the time*, with higher scores indicating higher levels of psychological resilience. The Cronbach’s α of CD-RISC in this study was 0.953.

### 2.3. Statistical analysis

Network analysis was performed using RStudio (version 4.1.1) software. The affect and emotion regulation-PsyCap network and affect and emotion regulation-psychological resilience network were constructed and visualized using the R package *qgraph* (69). A combination of least absolute shrinkage and selection operator (LASSO) regularization and extended Bayesian information criterion (EBIC) was applied to construct networks to compress trivial edges to zero (51, 70, 71). The EBIC hyperparameter was set to 0.5 to balance the sensitivity and specificity of the extraction of true edges (72). Spearman’s rho correlation was employed for network construction because of the ordinal nature of the data. In the two networks, nodes represent dimensions of affect, emotion regulation, PsyCap, and psychological resilience, while edges represent the partial correlation between two nodes after statistical control of the confounding influence of all other nodes in the network (73).

To identify the bridge nodes connecting communities, the node BEI was computed using the R package *networktools* (54). BEI is the sum of the cross-community edge weights of a given node (54) and is especially suitable for determining bridge nodes in a network with both positive and negative edges (74). A higher BEI indicates a greater impact of the bridge node on other communities and a higher likelihood that nodes of the other community are activated (52, 54). The nodes in each network were pre-divided into two communities, namely (i) the affect and emotion regulation community and (ii) the PsyCap or psychological resilience community.

The robustness of the two networks was tested by the R package *bootnet* (51), which ensures the accuracy and replicability of the network analysis. Firstly, the nonparametric bootstrap method (1,000 bootstrapped samples) was used to evaluate the accuracy of the edge weights by computing the 95% confidence interval (CI); the narrower the 95% CI, the more accurate the estimated edge weights (51, 75, 76). Next, the case-dropping bootstrap procedure (1,000 bootstrapped samples) was used to test the stability of the node BEI by calculating the correlation stability (CS) coefficient; a CS coefficient > 0.5 indicates ideal BEI stability (47). Finally, statistical differences between node BEIs and edge weights were examined by bootstrapped difference tests (1,000 bootstrapped samples, α = 0.05).

## 3. Results

### 3.1. Descriptive statistics

All participants were male. The sample included 391 (26.24%) sole offspring and 1,099 (73.76%) non-sole offspring; 1,315 (88.26%) from biparental and 175 (11.74%) from one-parent families; 737 (49.46%) had junior college education or below and 753 (50.54%) had a bachelor’s degree or above. The abbreviations, mean scores, and SDs for the variables selected in the present two networks are shown in Table 1.

**Table 1:** Abbreviations, means, and standard deviations (SDs) of each variable.

Variable	Abbreviation	Mean	SD
**Emotion regulation**			
Cognitive reappraisal	CR	28.29	7.05
Expressive suppression	ES	15.02	4.66
**Affect**			
Positive affect	POA	27.52	6.76
Negative affect	NEA	16.30	5.19
**Psychological capital**			
Self-efficacy	SEL	32.64	6.60
Resilience	RES	32.49	6.07
Hope	HOP	31.13	6.37
Optimism	OPT	31.00	6.74
**Psychological resilience**			
Tenacity	TEN	32.53	9.94
Strength	STR	22.18	5.97
Optimism	OP	8.72	3.07

### 3.2. Affect and emotion regulation-PsyCap network

**Figure 1:**
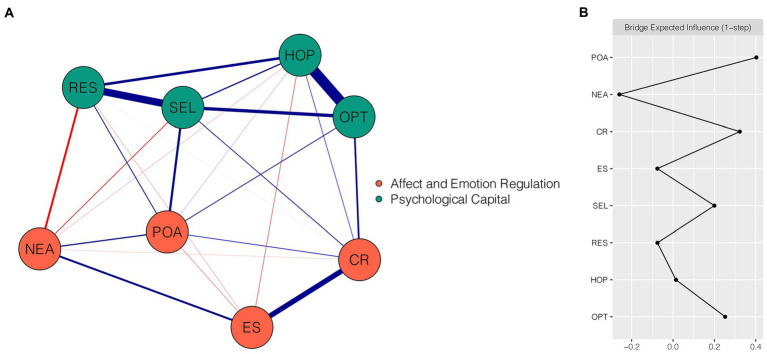
Network structure of emotion regulation, affect, and PsyCap dimensions. **(A)** EBICglasso network, where blue edges represent positive correlations and red edges represent negative correlations. A thicker edge reflects a higher correlation between nodes. **(B)** Centrality plot depicting the bridge expected influence of each node in the network (raw value). CR, cognitive reappraisal; ES, expressive suppression; POA, positive affect; NEA, negative affect; SEL, self-efficacy; RES, resilience; HOP, hope; OPT, optimism.

Figure 1A shows the final network of emotion regulation, affect, and PsyCap comprising 8 nodes and 24 non-zero edges (with weights ranging from −0.16 to 0.60) out of 28 possible edges. There were 11 within-community and 13 cross-community edges. Of the edges connecting the affect and emotion regulation community and the PsyCap community, relatively important edges were identified. Among these, POA “positive affect” was positively associated with SEL “self-efficacy” (weight = 0.18), RES “resilience” (weight = 0.11), and OPT “optimism” (weight = 0.10). NEA “negative affect” was negatively related to RES “resilience” (weight = −0.16). CR “cognitive reappraisal” exhibited positive associations with OPT “optimism” (weight = 0.15) and SEL “self-efficacy” (weight = 0.10). Supplementary Table S1 shows all edge weights within the affect and emotion regulation-PsyCap network. The bootstrapped 95% CI was narrow (see Supplementary Figure S1), suggesting that the edge weights had been accurately estimated. The bootstrapped difference test for edge weights in this network is shown in Supplementary Figure S2.

Figure 1B presents the raw BEI values for each node. In the affect and emotion regulation community, POA “positive affect” and CR “cognitive reappraisal” had the highest positive BEI values (BEI = 0.40 and 0.32, respectively), whereas NEA “negative affect” had the highest negative BEI value (BEI = −0.26). The CS coefficient of BEI was 0.75, indicating that the estimation of BEI was adequately stable (Supplementary Figure S3). Bootstrapped difference test showed that the BEI values of POA “positive affect,” CR “cognitive reappraisal,” and NEA “negative affect” were significantly different from those of 85.7–100% of other nodes (Supplementary Figure S4).

### 3.3. Affect and emotion regulation-psychological resilience network

**Figure 2:**
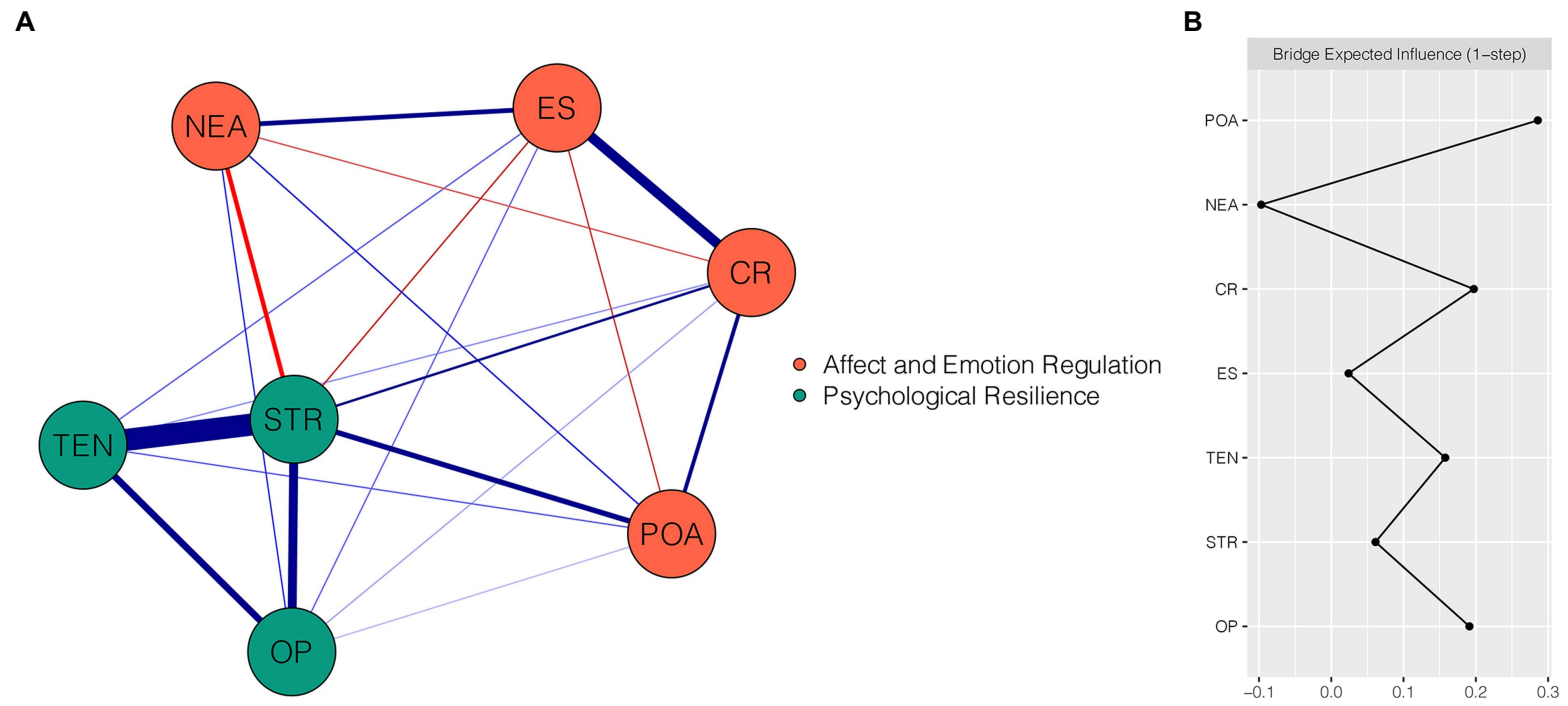
Network structure of emotion regulation, affect, and psychological resilience dimensions. **(A)** EBICglasso network, where blue edges represent positive correlations and red edges represent negative correlations. A thicker edge reflects a higher correlation between nodes. **(B)** Centrality plot depicting the bridge expected influence of each node in the network (raw value). CR, cognitive reappraisal; ES, expressive suppression; POA, positive affect; NEA, negative affect; TEN, tenacity; STR, strength; OP, optimism.

Figure 2A shows the final network of emotion regulation, affect, and psychological resilience, which comprises seven nodes. There were 20 non-zero edges (with edge weights ranging from −0.18 to 0.63) out of 21 possible edges, including 9 within-community edges and 11 cross-community edges. Among these cross-community edges, relatively strong edges were found. POA “positive affect” and CR “cognitive reappraisal” were positively linked with STR “strength” (weight = 0.20 and 0.12, respectively). NEA “negative affect” and ES “expressive suppression” were negatively linked with STR “strength” (weight = −0.18 and − 0.09, respectively). All edge weights within the present network are shown in Supplementary Table S2. The narrow bootstrapped 95% CI indicated that the edge weights of the network were accurate (Supplementary Figure S5). Supplementary Figure S6 presents the bootstrapped difference test results for edge weights.

Figure 2B depicts the raw BEI values for each node within the affect and emotion regulation-psychological resilience network. In the affect and emotion regulation community, POA “positive affect” and CR “cognitive reappraisal” exhibited the highest positive BEI values of 0.29 and 0.20, respectively, whereas NEA “negative affect” had the highest negative BEI value (BEI = −0.10). The CS coefficient of BEI was 0.75, which exceeded the preferred threshold and signified the stability of BEI (Supplementary Figure S7). The bootstrapped difference test showed that the BEI values of POA “positive affect,” CR “cognitive reappraisal,” and NEA “negative affect” were significantly different from those of 66.7–100% of other nodes (Supplementary Figure S8).

## 4. Discussion

The experience of the COVID-19 pandemic has dramatically impacted PsyCap and psychological resilience, especially among males. To identify protective and detrimental factors for PsyCap and resilience at a fine-grained level, this paper examined the network structure of emotion regulation, affect, and PsyCap, as well as the network structure of emotion regulation, affect, and resilience, among Chinese males during the late stage of the COVID-19 pandemic. The results showed that aspects of emotion regulation and affect function differently in relation to the dimensions of PsyCap and resilience, emphasizing certain relatively strong edges. Some of the dimensions were identified as bridge nodes that facilitate the impact of affect and emotion regulation on PsyCap and resilience. Importantly, these analyses support the accuracy and stability of the results.

It should be noted that certain strong cross-community edges were identified in the affect and emotion regulation-PsyCap network. POA “positive affect” was positively correlated with SEL “self-efficacy,” RES “resilience,” and OPT “optimism,” while NEA “negative affect” was negatively correlated with RES “resilience.” These results are consistent with a published study reporting that positive affect and PsyCap are positively associated, and that the opposite is true for the relation between negative affect and PsyCap (32). Other previous studies have also found that positive affect exerts a positive effect on PsyCap (77, 78). In the fine-grained exploration of the pathways between affect and PsyCap carried out in the present study, positive affect was associated with self-efficacy. This is consistent with previous reports showing that positive affect is a significant predictor of self-efficacy and that positive affect is a moderator between personal accomplishment and creative self-efficacy (79–81). Regarding positive affect and resilience, many lines of evidence support their close relationship and reciprocal reinforcement effect (82–84). It has even been suggested that positive affect may be part of resilience in a broad sense (85). Considering the positive correlation between positive affect and resilience, it was not surprising that negative affect was negatively associated with resilience, which is consistent with previous research (86, 87). Furthermore, a positive edge was found between positive affect and optimism, which is reasonable given their similar meanings (88). This finding is also in line with previous studies (89, 90).

Two further relatively strong cross-community edges were identified in the affect and emotion regulation-PsyCap network. CR “cognitive reappraisal” was positively associated with OPT “optimism” and SEL “self-efficacy,” implying that frequent use of a cognitive reappraisal strategy will likely increase PsyCap. Existing research has shown an intimate relationship between cognitive reappraisal and optimism. These two variables are regarded as components of personal resources or a hopeful future orientation, and lower levels of cognitive reappraisal and optimism were shown to contribute to higher psychological distress during COVID-19; predictably, there may be a close relation between these two variables (91, 92). Cognitive reappraisal represents a strategy of reinterpreting an emotion-eliciting situation in a way that reduces its negative impact (93–95), which can partly explain the relation between cognitive reappraisal and optimism. The positive link between cognitive reappraisal and self-efficacy is consistent with previous studies, reporting that cognitive reappraisal fosters anticipatory psychological appraisal of self-efficacy and greater self-efficacy and control under stress (96, 97). Importantly, cognitive reappraisers are predicted to be more optimistic and to have a greater sense of self-efficacy in regard to their immediate environment (63), which is consistent with the results of the present study.

In the affect and emotion regulation-psychological resilience network, certain relatively strong cross-community edges were found. STR “strength” was positively correlated with POA “positive affect” and CR “cognitive reappraisal,” but negatively correlated with NEA “negative affect” and ES “expressive suppression.” In general, previous studies have found that resilience is positively correlated with and fueled by positive affect and negatively correlated with negative affect (33–35), and that emotion expression and cognitive reappraisal can enhance psychological resilience (98, 99). These findings further suggest that affect and emotion regulation relate to psychological resilience at the dimension level—a relationship that is commonly overlooked by previous sum-score analyses. A previous correlational study found that the positive affect score was positively associated with the strength dimension of resilience, but the opposite was true for negative affect (100); these results are in line with those of the current study. There are several possible explanations for the links between emotion regulation strategies (i.e., cognitive reappraisal and expressive suppression) and strength. For example, cognitive reappraisal involves changing the way to think about a challenging situation, which may facilitate subjective perceptions of strength of psychological resilience. In contrast, expressive suppression involves hiding and inhibiting outward emotional expression, which results in an accumulation of negative emotions and undermines mental well-being, and can lead to the development of anxiety and depression during the late stage of COVID-19 (55, 101); hence, the strength dimension of psychological resilience may also be negatively impacted. Given that there are no studies with which to compare the findings of the present work, this issue should be validated in the future.

To quantify the impact of the dimensions of affect or emotion regulation on PsyCap and psychological resilience, the BEI of each node in the respective network was calculated. However, the BEI values of nodes in the affect and emotion regulation community were most intriguing. In the two constructed networks, POA “positive affect,” CR “cognitive reappraisal,” and NEA “negative affect” were identified as critical bridge nodes. Positive affect and cognitive reappraisal exhibited positive BEI values, indicating their beneficial effects on PsyCap and psychological resilience; in contrast, negative affect had a negative BEI value and may be a detrimental factor for PsyCap and resilience. As mentioned above, the three nodes were directly connected with the dimensions of PsyCap and psychological resilience. These findings are consistent with previous studies reporting that positive affect and cognitive reappraisal have positive effects, whereas negative affect exerts adverse effects, on PsyCap and psychological resilience (32–35, 77, 78, 98, 99). The present study adds further evidence for this from a network-theory perspective.

These findings have important implications. Regarding theoretical implications, examining the fine-grained relationships between affect or emotion regulation and PsyCap and psychological resilience provides preliminary insights into the specific pathways linking these psychological constructs. The active interactions between these dimensions, such as the relationship between positive affect and self-efficacy, facilitate the understanding of the mechanisms underlying the protective and risk-related roles of affect and emotion regulation for PsyCap and psychological resilience. Regarding practical implications, bridge nodes play important roles in the co-occurrence of psychological constructs and promote the transmission of positive or negative influences of one community on another (54). Hence, from the network analysis perspective, critical bridge nodes are potential targets for intervention (52, 54–56, 102). In the current study, positive affect, cognitive reappraisal, and negative affect are critical bridge nodes and thus are suggested as potential intervention targets, providing implications for clinical care and public mental health. For example, greater experience of positive affect, attenuation of negative affect, and frequent use of cognitive reappraisal could contribute to enhancing PsyCap and psychological resilience. Thus, this study offers meaningful theoretical and practical implications for the mental health of males in the context of the COVID-19 pandemic. Moreover, the new information provided by these findings can also be applied to other epidemics that show similarities to COVID-19 to a certain extent. This practice has been employed by other studies (103–105). For instance, a study suggested that the psychological intervention measures employed during the COVID-19 pandemic would be applicable to similar future epidemics (103); other studies drew on evidence from previous coronavirus outbreaks, namely severe acute respiratory syndrome (SARS) and Middle East Respiratory syndrome (MERS), to preliminarily obtain information regarding the psychological or neuropsychiatric implications of the COVID-19 pandemic (104, 105). Therefore, the findings of the present study can also be used as a reference for similar epidemics in the future.

The strengths of this study include its multi-center study design, large sample size, and utilization of network analysis with stable results. However, as with any research, this study is subject to limitations, which provide avenues for future research. First, the sample only included male adults, which limits the generalizability of the findings. Future studies should verify the extension of these findings to other populations such as females or the elderly. Second, because of the cross-sectional design of this study, causality between the dimensions of different constructs cannot be inferred. Future research should examine temporal causal relationships using longitudinal or experimental designs. Third, affect, emotion regulation, PsyCap, and psychological resilience were measured using self-report scales, which are predisposed to subjective bias. Thus, all results should be interpreted with caution. Fourth, as certain relevant aspects were not captured because only one scale was used to assess each psychological construct, the present study can only provide preliminary insights into the examined relationships. Future studies are encouraged to include other aspects of these constructs to conduct comprehensive examinations. Finally, because of resource-related reasons, selection bias cannot be ruled out as random sampling was not employed when recruiting participants.

## 5. Conclusion

This is the first study that uses network analysis to better understand the dimension-level interrelations between emotion regulation or affect and PsyCap and psychological resilience among Chinese males during the late stage of the COVID-19 pandemic. The findings elucidate the specific pathways through which these dimensions interact with aspects of PsyCap and psychological resilience. These pathways emphasize the positive roles of positive affect and cognitive reappraisal and the detrimental role of negative affect. These results have implications for clinical care and public mental health and provide references for targeted intervention strategies to enhance PsyCap and psychological resilience. This reference provides a basis for attenuating the adverse effects the COVID-19 pandemic imposed on mental well-being.

## Data availability statement

The raw data supporting the conclusions of this article will be made available by the authors, without undue reservation.

## Ethics statement

The studies involving human participants were reviewed and approved by the Ethics Committee of the First Affiliated Hospital of the Air Force Medical University. The patients/participants provided their written informed consent to participate in this study.

## Author contributions

ZG, YZ, and XZ: conceptualization. TY, YC, XL, and HL: formal analysis and investigation. ZG, YC, and TY: writing—original draft preparation. YZ and XZ: writing—review and editing. XZ: funding acquisition. All authors contributed to the article and approved the submitted version.

## Funding

This work was supported by the Major Project of Medicine Science and Technology of PLA (AWS17J012).

## Conflict of interest

The authors declare that the research was conducted in the absence of any commercial or financial relationships that could be construed as a potential conflict of interest.

## Publisher’s note

All claims expressed in this article are solely those of the authors and do not necessarily represent those of their affiliated organizations, or those of the publisher, the editors and the reviewers. Any product that may be evaluated in this article, or claim that may be made by its manufacturer, is not guaranteed or endorsed by the publisher.
