# Supplementary material for: Network analysis of affect, emotion regulation, psychological capital, and resilience among Chinese males during the late stage of the COVID-19 pandemic
Source: Front Public Health. 2023 Mar 27;11:1144420. doi: 10.3389/fpubh.2023.1144420 (PMC10083324; doi:10.3389/fpubh.2023.1144420)
Supplement: Supplementary file 1 [file Data_Sheet_1.docx]

Supplementary Material

Supplementary Table 1 All the edge weights in the affect and emotion regulation-PsyCap network

|  | POA | NEA | CR | ES | SEL | RES | HOP | OPT |
| --- | --- | --- | --- | --- | --- | --- | --- | --- |
| POA | 0 |  |  |  |  |  |  |  |
| NEA | 0.12 | 0 |  |  |  |  |  |  |
| CR | 0.09 | -0.01 | 0 |  |  |  |  |  |
| ES | -0.04 | 0.16 | 0.33 | 0 |  |  |  |  |
| SEL | 0.18 | -0.08 | 0.10 | 0 | 0 |  |  |  |
| RES | 0.11 | -0.16 | 0.004 | -0.02 | 0.44 | 0 |  |  |
| HOP | 0.02 | -0.02 | 0.07 | -0.05 | 0.12 | 0.19 | 0 |  |
| OPT | 0.10 | 0 | 0.15 | 0 | 0.23 | 0 | 0.60 | 0 |

*Note*: POA, positive affect; NEA, negative affect; CR, cognitive reappraisal; ES, expressive suppression; SEL, self-efficacy; RES, resilience; HOP, hope; OPT, optimism.

Supplementary Figure 1 Accuracy of edge weights in the affect and emotion regulation-PsyCap network

*Note*: The red line depicts the sample edge weights and the gray bar depicts the bootstrapped confidence interval. POA, positive affect; NEA, negative affect; CR, cognitive reappraisal; ES, expressive suppression; SEL, self-efficacy; RES, resilience; HOP, hope; OPT, optimism.

Supplementary Figure 2 Bootstrapped difference test for edge weights in the affect and emotion regulation-PsyCap network

*Note*: Gray boxes indicate edge weights that do not differ significantly from one another, while black boxes indicate edge weights that do differ significantly. Blue and red boxes on the diagonal correspond to edge weights with positive and negative correlations, respectively. POA, positive affect; NEA, negative affect; CR, cognitive reappraisal; ES, expressive suppression; SEL, self-efficacy; RES, resilience; HOP, hope; OPT, optimism.

Supplementary Figure 3 Stability of node bridge expected influences in the affect and emotion regulation-PsyCap network

*Note*: The red bar represents the average correlation between node bridge expected influences in the full sample and subsample with the red area depicting the 2.5th quantile to the 97.5th quantile.

Supplementary Figure 4 Bootstrapped difference test for node bridge expected influences in the affect and emotion regulation-PsyCap network

*Note*: Gray boxes indicate node bridge expected influences that do not differ significantly from one another, while black boxes indicate node bridge expected influences that do differ significantly. POA, positive affect; NEA, negative affect; CR, cognitive reappraisal; ES, expressive suppression; SEL, self-efficacy; RES, resilience; HOP, hope; OPT, optimism.

Supplementary Table 2 All the edge weights in the affect and emotion regulation-psychological resilience network

|  | POA | NEA | CR | ES | TEN | STR | OP |
| --- | --- | --- | --- | --- | --- | --- | --- |
| POA | 0 |  |  |  |  |  |  |
| NEA | 0.10 | 0 |  |  |  |  |  |
| CR | 0.16 | -0.06 | 0 |  |  |  |  |
| ES | -0.08 | 0.18 | 0.32 | 0 |  |  |  |
| TEN | 0.06 | 0 | 0.04 | 0.06 | 0 |  |  |
| STR | 0.20 | -0.18 | 0.12 | -0.09 | 0.63 | 0 |  |
| OP | 0.02 | 0.08 | 0.03 | 0.06 | 0.24 | 0.29 | 0 |

*Note*: POA, positive affect; NEA, negative affect; CR, cognitive reappraisal; ES, expressive suppression; TEN, tenacity; STR, strength; OP, optimism.

Supplementary Figure 5 Accuracy of edge weights in the affect and emotion regulation-psychological resilience network

*Note*: The red line depicts the sample edge weights and the gray bar depicts the bootstrapped confidence interval. POA, positive affect; NEA, negative affect; CR, cognitive reappraisal; ES, expressive suppression; TEN, tenacity; STR, strength; OP, optimism.

Supplementary Figure 6 Bootstrapped difference test for edge weights in the affect and emotion regulation-psychological resilience network

*Note*: Gray boxes indicate edge weights that do not differ significantly from one another, while black boxes indicate edge weights that do differ significantly. Blue and red boxes on the diagonal correspond to edge weights with positive and negative correlations, respectively. POA, positive affect; NEA, negative affect; CR, cognitive reappraisal; ES, expressive suppression; TEN, tenacity; STR, strength; OP, optimism.

Supplementary Figure 7 Stability of node bridge expected influences in the affect and emotion regulation-psychological resilience network

*Note*: The red bar represents the average correlation between node bridge expected influences in the full sample and subsample with the red area depicting the 2.5th quantile to the 97.5th quantile.

Supplementary Figure 8 Bootstrapped difference test for node bridge expected influences in the affect and emotion regulation-psychological resilience network

*Note*: Gray boxes indicate node bridge expected influences that do not differ significantly from one another, while black boxes indicate node bridge expected influences that do differ significantly. POA, positive affect; NEA, negative affect; CR, cognitive reappraisal; ES, expressive suppression; TEN, tenacity; STR, strength; OP, optimism.
